# Evidence for a protective effect of the loss of α4-containing nicotinic acetylcholine receptors on Aβ-related neuropathology in Tg2576 mice

**DOI:** 10.3389/fnins.2023.1097857

**Published:** 2023-04-11

**Authors:** Antonietta Vilella, Benedetto Romoli, Martina Bodria, Stéphanie Pons, Uwe Maskos, Michele Zoli

**Affiliations:** ^1^Department of Biomedical, Metabolic and Neural Sciences, Center for Neuroscience and Neurotechnology, University of Modena and Reggio Emilia, Modena, Italy; ^2^Institut Pasteur, Université Paris Cité, Neurobiologie Intégrative des Systèmes Cholinergiques, CNRS UMR 3571, Département de Neuroscience, Paris, France

**Keywords:** α4-containing nicotinic acetylcholine receptors, microglia, Alzheimer’s disease, Tg2576, APPswe

## Abstract

**Introduction:**

Loss of cholinergic neurons as well as α4β2* (* = containing) nicotinic acetylcholine receptors (nAChRs) is a prominent feature of Alzheimer’s disease (AD). Specifically, amyloid β (Aβ), the principal pathogenic factor of AD, is a high affinity ligand for nAChRs. Yet, the pathophysiological role of nAChRs in AD is not well established.

**Methods:**

In the present study, we have investigated the effects of the loss of α4* nAChRs on the histological alterations of the Tg2576 mouse model of AD (APPswe) crossing hemizygous APPswe mice with mice carrying the genetic inactivation of α4 nAChR subunit (α4KO).

**Results:**

A global decrease in Aβ plaque load was observed in the forebrain of APPswe/α4KO mice in comparison with APPswe mice, that was particularly marked in neocortex of 15 month-old mice. At the same age, several alterations in synaptophysin immunoreactivity were observed in cortico-hippocampal regions of APPswe mice that were partially counteracted by α4KO. The analysis of the immunoreactivity of specific astroglia (glial fibrillary acidic protein, GFAP) and microglia (ionized calcium-binding adapter molecule, Iba1) markers showed an increase in the number as well as in the area occupied by these cells in APPswe mice that were partially counteracted by α4KO.

**Conclusion:**

Overall, the present histological study points to a detrimental role of α4* nAChRs that may be specific for Aβ-related neuropathology.

## 1. Introduction

Neuronal nicotinic acetylcholine receptors (nAChRs) are composed of 5 subunits belonging to a superfamily of proteins subdivided into α2-10 and β2-4 subunits. Although multiple subtypes of nAChRs are expressed in the mammalian brain, the majority is represented by the heteromeric α4β2* (* = containing) subtype with high affinity for nicotine, and the homomeric α7 subtype with low affinity for nicotine and high affinity for α-bungarotoxin. Minor nAChR subtypes include α3β2/β4* and α6β2/β4* as well as α7β2 nAChRs (Moretti et al., 2014; Zoli et al., 2015). Among their multiple roles, a number of studies have shown an involvement of nAChRs in neuropsychiatric disorders, such as autism and schizophrenia, and in age-related neurodegenerative diseases such as Alzheimer’s disease (AD) and Parkinson’s disease (PD; Picciotto and Zoli, 2008; Schliebs and Arendt, 2011; Lombardo and Maskos, 2015).

Several lines of evidence indicate that nAChRs may exert neuroprotective effects. In fact, direct stimulation of nAChRs with nicotine or other nicotinic agonists elicits neuroprotective effects in animal models both *in vitro* and *in vivo* [reviewed in Picciotto and Zoli, 2008] and smoking is associated with lower incidence of PD though not of AD (Li et al., 2015), although nicotine patches are in clinical trials for mild cognitive impairment (Newhouse et al., 2012). In addition to neuroprotective actions, nicotine can exert anti-inflammatory effects through α7 nAChRs expressed by monocytes/macrophages or microglia (Noda and Kobayashi, 2017). Finally, a marked and consistent decrease in high affinity nicotine binding and α4β2* receptors in cortico-hippocampal regions have been demonstrated in several forms of dementia (Gotti et al., 2006a; Wu et al., 2010; Teipel et al., 2011; Bekdash, 2021). Accordingly, knockout (KO) mice lacking β2* nAChRs (Picciotto et al., 1995; Zoli et al., 1998) spontaneously develop neuronal loss and gliosis in cortical areas and cognitive deficits during senescence (Zoli et al., 1999; Huang et al., 2011) and have increased cognitive impairments and neuronal loss after an excitotoxic lesion of the hippocampal formation (Zanardi et al., 2007). Overall, these data suggest that loss of β2* nAChRs removes a level of protection against neurodegenerative processes associated with senescence and some types of insult.

Loss of cholinergic neurons and terminals in basal forebrain and cortex, respectively, as well as nAChR loss (see above) are prominent features of AD (Hellstrom-Lindahl and Court, 2000; Gotti et al., 2006b; Hoskin et al., 2019; Bekdash, 2021), especially of the early phase of the disease, and stimulation of cholinergic function using acetylcholine esterase inhibitors is an approved therapeutic strategy for AD. Yet, the pathophysiological role of nAChRs in AD is not well established and may have peculiar features in relation to amyloid β (Aβ)-associated neuropathology.

In fact, Aβ, a key player in the development of AD, can bind α7 nAChRs with picomolar affinity and α4β2 nAChRs with nanomolar affinity (Puzzo et al., 2008, 2011; Fabiani and Antollini, 2019; Roberts et al., 2021). Indeed, Aβ-induced neuronal dysfunctions, such as reduction of AMPA receptor phosphorylation and surface expression, and disruption of glycine-elicited long-term potentiation (LTP) in hippocampal cultures, were shown to be reversed by the co-activation of α7- and α4β2-nAChRs (Roberts et al., 2021).

The interaction between Aβ and α7 nAChRs has been extensively investigated and is thought to mediate physiological effects of Aβ at low concentration and Aβ neurotoxicity at higher concentrations, possibly through internalization of the nAChR/Aβ complex (Farhat and Ahmed, 2017; Gulisano et al., 2019). This interaction may mediate part of the neural dysfunctions of the early stages of AD (Roberts et al., 2021; Tropea et al., 2021).

In general, much less is known on the specific impact of α4β2 nAChRs on Aβ-related pathologies. Interestingly, a recent paper strongly suggested a detrimental impact of β2* nAChRs on Aβ-induced neurotoxicity. In fact, hAPP-SLA (a lentiviral vector encoding the human sequence of amyloid precursor protein, hAPP, harboring the *S*wedish, *L*ondon and *A*ustrian pathogenic mutations) delivered into the dentate gyrus of β2* KO mice, induced decreased intracellular Aβ accumulation in dentate gyrus and reduced impairment in recognition memory with respect to control mice (Lombardo et al., 2016). As a possible mechanistic counterpart of this *in vivo* evidence, it has been shown that expression of a α4β2 nAChRs sensitizes the neurotoxicity elicited by oligomeric Aβ (Arora et al., 2015, 2020).

In the present study, we have investigated the effects of the loss of α4* nAChRs on the histological alterations of Tg2576 mice, that overexpress human amyloid precursor protein (hAPP) with the Swedish mutation (KM670/671NL, APPswe; Kosel et al., 2020).

## 2. Materials and methods

### 2.1. Animals

Experimental animals were generated by crossing hemizygous Tg2576 mice (Taconic Biosciences, Rensselaer, NY, United States) expressing APPswe with α4^−/−^ mice (Charles River France; Marubio et al., 1999). Tg2576 mice were crossed with α4^−/−^ breeders to obtain Tg2576/α4^+/−^ mice, that, in turn, were crossed with α4^+/−^ to obtain the four experimental groups: APPswe^−/−^/α4^+/+^ control (Ctrl) mice, APPswe^−/−^/α4^−/−^ (α4KO) mice, APPswe^+/−^/α4^+/+^ (APPswe) mice, APPswe^+/−^/α4^−/−^ (APPswe/α4KO) mice (C57Bl6J background). Male and female 5 and 15 month-old (mo) mice were used. Mice were kept in conditioned rooms with stable temperature (21°C) and humidity (60%), on a light/dark cycle of 12 h. Food and water were available *ad libitum* and body weight was recorded throughout the entire observation period. All animal procedures were approved by the Committee on Animal Health and Care of the University of Modena and Reggio Emilia (protocol number: 102/2011-B) and conducted in accordance with National Institutes of Health guidelines.

### 2.2. Histological procedures

Mice were anesthetized with isoflurane and sacrificed by intracardiac perfusion with cold 4% paraformaldehyde (PFA) preceded by an infusion of 50 mL of 0.9% NaCl saline containing heparin sodium (5,000 U/L); the brains were postfixed in the same solution for 12 h, rinsed in 15% sucrose in PBS for approximately 12 h and then in 30% sucrose in PBS for 1 day. Fixed brains were cut at the cryostat (20 μm thickness) and processed according to established protocols to test cell-specific AD-like alterations through immunohistochemical (IHC) analysis (Daini et al., 2022).

The following antibodies (Abs) were used: rabbit anti-human APP (1:1,000, Cell signaling, #2452), mouse anti-human Aβ/APP 6E10 (epitope human Aβ1-16, 1:500, Signet, #9300-10), mouse anti-human Aβ MoAb2 (epitope recombinant human Aβ oligomers, 1:500, Millipore, #MABN254), mouse anti-human Aβ 11A1 (epitope synthetic peptide of E22P-Aβ 10–35 part, 1:500, Tecan, #10379), rabbit anti-Ionized calcium-binding adapter molecule 1 (Iba1, 1:1,000, Wako, #019-19,741), rabbit anti-glial fibrillary acidic protein (GFAP, 1:2,000, Dako, #Z0334) and rabbit anti-synaptophysin (SYN, 1:1,000, SYSY, #101004). A pre-treatment with formic acid was performed for anti-Aβ Abs when specified by the manufacturer. Vectastain ABC-HRP (#PK-4002; #PK-4001) kits were used for peroxidase diaminobenzidine staining; slices were placed on gelatinized glass slides, dehydrated and mounted with Eukitt® mounting medium. Congo Red (optical and fluorescent dye with high affinity for the *β-sheet* structure and thus marker for fibrillar Aβ) and Nissl stainings were performed according to established protocols (Daini et al., 2022).

### 2.3. Sampling and image analysis

Immunolabeled brain slices were photographed on a Nikon Eclipse CiL microscope (4×, 10×, or 40× objective) through a Nikon DS-Fi3 camera under constant light conditions. All evaluations were performed on coded slides by at least two experimenters.

The semi-quantitative densitometric (i.e., specific optical density) and morphometric (i.e., count, areas, thickness, length) analyses were performed following established protocols (Zoli et al., 1990, 1992; Daini et al., 2022) using routines from the Nikon NIS-Elements D (5.21.00v) and ImageJ Fiji programs.

Congo Red- or Aβ-immunoreactive plaque analysis was performed on images obtained with a 20x objective. Plaques were manually selected within the neocortex (nCtx; Bregma between −0.7 and −0.8 mm), the entorhinal cortex (Ent; Bregma between −3.1 and −3.2 mm) and the hippocampus (Hip; Bregma between −1.9 and −2.2 mm). First, images were manually edited to remove edge artifacts, folds, and blood vessels, then subjectively thresholded. To quantify global amyloid plaque extension, the % positive area covered by all amyloid plaques within the manually outlined regions of interest (Ctx, Hip) was automatically obtained through ImageJ software. Other quantitative morphometric parameters such as plaque number, area and perimeter of every plaque in the analyzed areas, were measured through a Nikon NIS-Elements D software.

In the analysis of Nissl staining, at least three somatosensory cortex (sCtx) and Hip slices/mouse (Bregma between −0.7 and −0.8 mm and between −1.9 and −2.2 mm, respectively) were acquired with 4× objective and layer thickness quantified through the *length/thickness* function of ImageJ software.

For SYN immunoreactivity (ir) analysis, three slices/mouse from sCtx and Hip (Bregma between −0.7 and −0.8 mm and between −1.9 and −2.2 mm, respectively) were acquired with 10× objective and the morphometric parameters quantified through the *length/thickness* and *area* functions of ImageJ software. For densitometric analysis, acquired images were converted to 32 bit grayscale and specific optical density values were obtained by subtracting the optical density of the sampled region from the optical density of non-specific staining, i.e., corpus callosum for both cortex and CA3 analysis.

For densitometric analysis of GFAP and Iba1 immunostainings, at least three slices/mouse from cingulate cortex (cCtx; Bregma between −0.5 and −0.9), sCtx (Bregma between −0.7 and −0.8 mm) and Hip (Bregma between −1.9 and −2.2 mm) were acquired with 10× objective. Acquired images were converted to 32 bit grayscale and a thresholding procedure was applied (−20 units from the peak mean gray value) and % positive area, i.e., the area covered by pixels with gray value above the threshold, was automatically recorded by means of *analyze particles* function of ImageJ software.

As specifically concerns the analysis of morphometric parameters related to microglia, firstly Iba1+ cell count was performed manually, in live mode, within the area of interest by using a 40× objective; in the same section, for each Iba1+ cell, cell outline was manually selected and stereological parameters, such as area, perimeter, number of primary processes and total primary process length, were automatically recorded by means of Nikon NIS-Elements D software. Finally, microglial phenotyping and characterization was performed on the basis of the number of processes and soma volume as describe in the literature with minor changes (Franco-Bocanegra et al., 2021). Briefly, cell phenotype was attributed on the basis of the number and morphology of processes: cells with 5 or more long, thin, highly branched processes were classified as homeostatic cells, cells with reduced number (3–4) of unbranched or less branched shortened processes were classified as less ramified cells and, cells without processes or 1–2 shortened and unbranched processes were classified as amoeboid cells (Torres-Platas et al., 2014; Franco-Bocanegra et al., 2021).

### 2.4. Statistical analysis

Values are shown as mean ± standard error of the mean (SEM) or 95% confidence interval as appropriate. Group differences were analyzed by means of two-way ANOVA or Mann–Whitney *U*-test as appropriate. Proportions were analyzed by means of the Chi square test. Correlations were analyzed by means of the Spearman test. Statistical analyses were performed through SPSS software, with *p* < 0.05 as the level for a significant difference and 0.10 > *p* > 0.05 as trend for a significant difference.

## 3. Results

Development of AD-like morphological alterations in Tg2576 mouse brains concerns deposition of Aβ and associated neural tissue alterations. In this context, we sought to characterize the possible effects of α4* nAChR loss in AD-like morphological alterations using a quantitative analysis of several relevant histological parameters: plaques were characterized by their content of amyloid proteins (Congo red) and immunohistochemical (IHC) staining for several Aβ forms. Overall atrophy of neural tissue was analyzed through Nissl staining, while specific changes in density and morphology of reactive cells, astro- and micro-glia, were studied by means of GFAP and Iba1 IHC staining, respectively, and global changes in synaptic structures by means of IHC staining for SYN.

### 3.1. Amyloid plaques

In order to assess whether the absence of α4* nAChRs influences Aβ plaque load, coronal brain slices from APPswe^−/−^/α4^+/+^ (Ctrl), APPswe^−/−^/α4^−/−^ (α4KO), APPswe^+/−^/α4^+/+^ (APPswe), and APPswe^+/−^/α4^−/−^ (APPswe/α4KO) mice were processed by means of Congo Red standard stain (for amyloidosis detection) and IHC staining with different Abs for unaggregated, oligomeric, and fibrillar forms of human Aβ42 and unaggregated Aβ40 (anti-MoAb2 Ab) or Aβ42 neurotoxic oligomers (anti-11A1 Ab).

In 5 mo mice, no Congo Red, MoAb2+ or 11A1+ plaques were detected in any mouse group including APPswe and APPswe/α4KO. Interestingly, an anti-human APP Ab showed intracellular staining in APPswe mice which was similarly intense in APPswe/α4KO mice (Mann–Whitney *U*-test, *p* = 0.70), and was absent in Ctrl and α4KO mice (Supplementary Figure 1).

**Figure 1:**
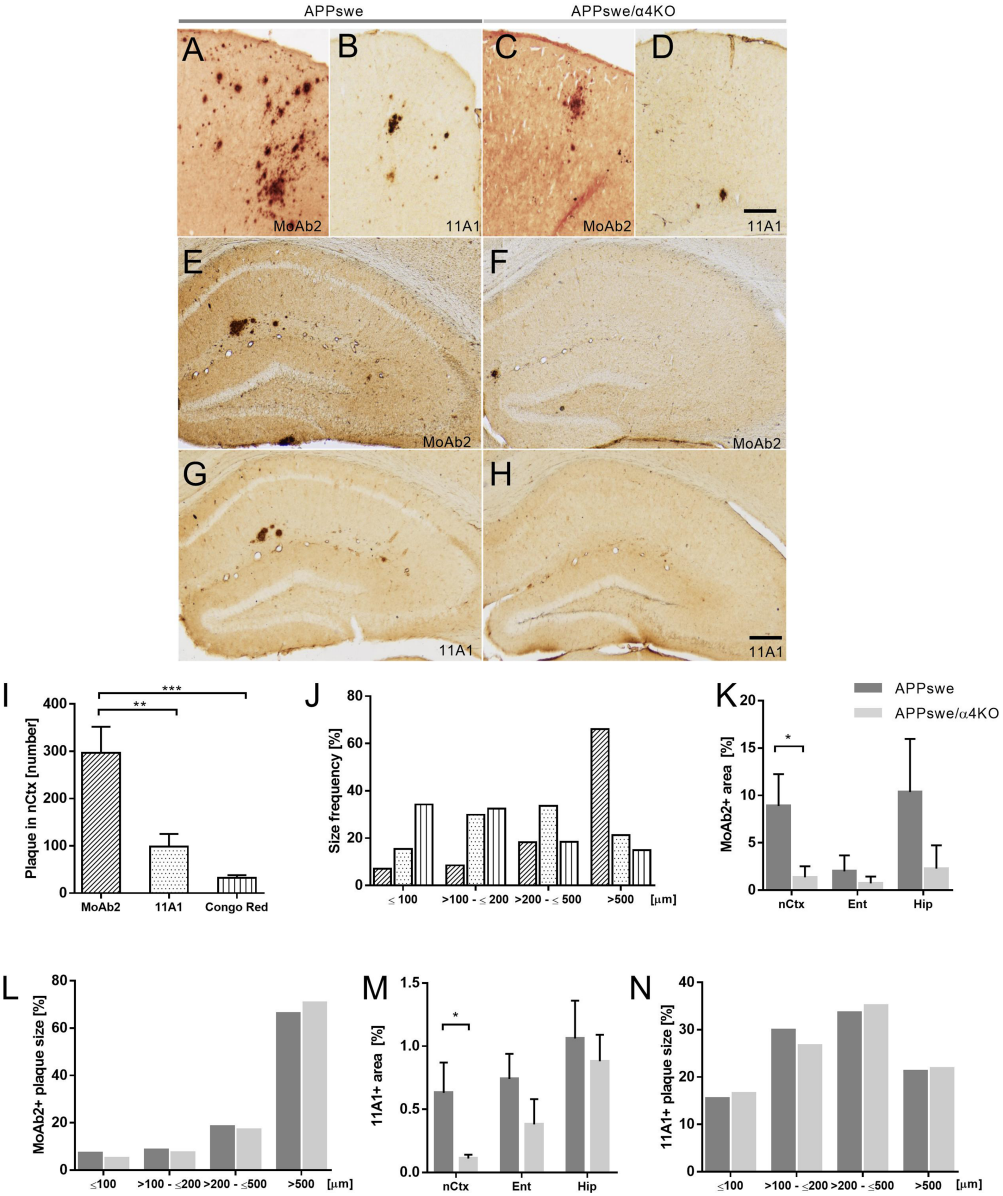
Amyloid plaque characterization and distribution in APPswe and APPswe/α4KO mice. **(A–D)** Representative images of MoAb2+ **(A,C)** and 11A1+ plaques **(B,D)** in the nCtx of APPswe **(A,B)** and APPswe/α4KO **(C,D)** 15 mo mice. **(E–H)** Representative images of MoAb2+ and 11A1+ plaques in the Hip of 15 mo APPswe **(E,G)**, and APPswe/α4KO **(F,H)** mice. Scale bar = 100 μm. **(I,J)** Congo Red+, MoAb2+, and 11A1+ plaque count **(I)** and size distribution **(J)** in the nCtx of APPswe mice (^**^p < 0.01, ^***^p < 0.001). **(K,N)** Analysis of MoAb2+ plaque area in nCtx, Ent and Hip of APPswe and APPswe/α4KO mice (^*^p < 0.05). **(L,N)** MoAb2+ and **(N)** 11A1+ plaque size distribution in nCtx of APPswe (dark gray bar) and APPswe/α4KO (gray bar) mice. In **I,K,L**, data are shown as mean *±* SEM and compared by one-way ANOVA followed by Bonferroni correction **(I)** and by Mann–Whitney *U*-test **(K,M)**. In **J,L,N**, frequencies are graphed as % by each group and compared by Chi square test. nCtx, neocortex; Ent, entorhinal cortex; Hip, hippocampal formation.

In 15 mo mice, amyloid plaques were detected in both APPswe and APPswe/α4KO but never in Ctrl or α4KO mice. To characterize amyloid deposits, we performed a morphometric analysis of plaques labeled with MoAb2 and 11A1 Abs or Congo Red stain (not shown). Figure 1 provides representative images of cortical and hippocampal MoAb2+ (Figures 1A,C,E,F, respectively) and 11A1+ (Figures 1B,D,G,H, respectively) amyloid plaques in 15 mo mice (Figures 1A–H).

Our analysis demonstrated that in APPswe mice, while the regional distribution of Congo Red, MoAb2+ and 11A1+ plaques was comparable, the morphometric features of plaques were staining-specific. The number and size of MoAb2+ plaques were much larger than those of 11A1+ plaques which were, in turn, more numerous and larger than Congo Red stained plaques (Figures 1I,J). Indeed, while 11A1+ plaque number was highly significantly correlated with the number of Congo Red stained plaques in the nCtx of APPswe mice (Spearman Rho = 0.857, *p* = 0.007, n = 8), the number of MoAb2+ plaques was not correlated with the number either of 11A1+ or of Congo Red plaques (not shown).

A global decrease in both MoAb2+ and 11A1+ plaque number/sampled region was observed in the forebrain of APPswe/α4KO mice in comparison with APPswe mice, that was particularly marked in nCtx (Figures 1K,L). The distribution of plaque size was, however, comparable in the two mouse groups (chi square test, MoAb2, *p* = 0.1254, 11A1, *p* = 0.9419; Figures 1M,N).

These results demonstrate a decrease in the number of amyloid deposits associated with the maintenance of size distribution suggesting that the main effect of α4KO is on plaque production since the process of their growth is substantially maintained. Detailed statistical reports are shown in Supplementary Table 1.

### 3.2. Atrophy of cerebral cortex and hippocampus

Since amyloid-related dendritic atrophy is described in AD and neuronal populations of the primary sCtx are severely affected in the Tg2576 mouse model of AD (Somogyi et al., 2016), we analyzed the thickness of neuronal layers in Nissl-stained sCtx as a marker of tissue atrophy.

**Figure 2:**
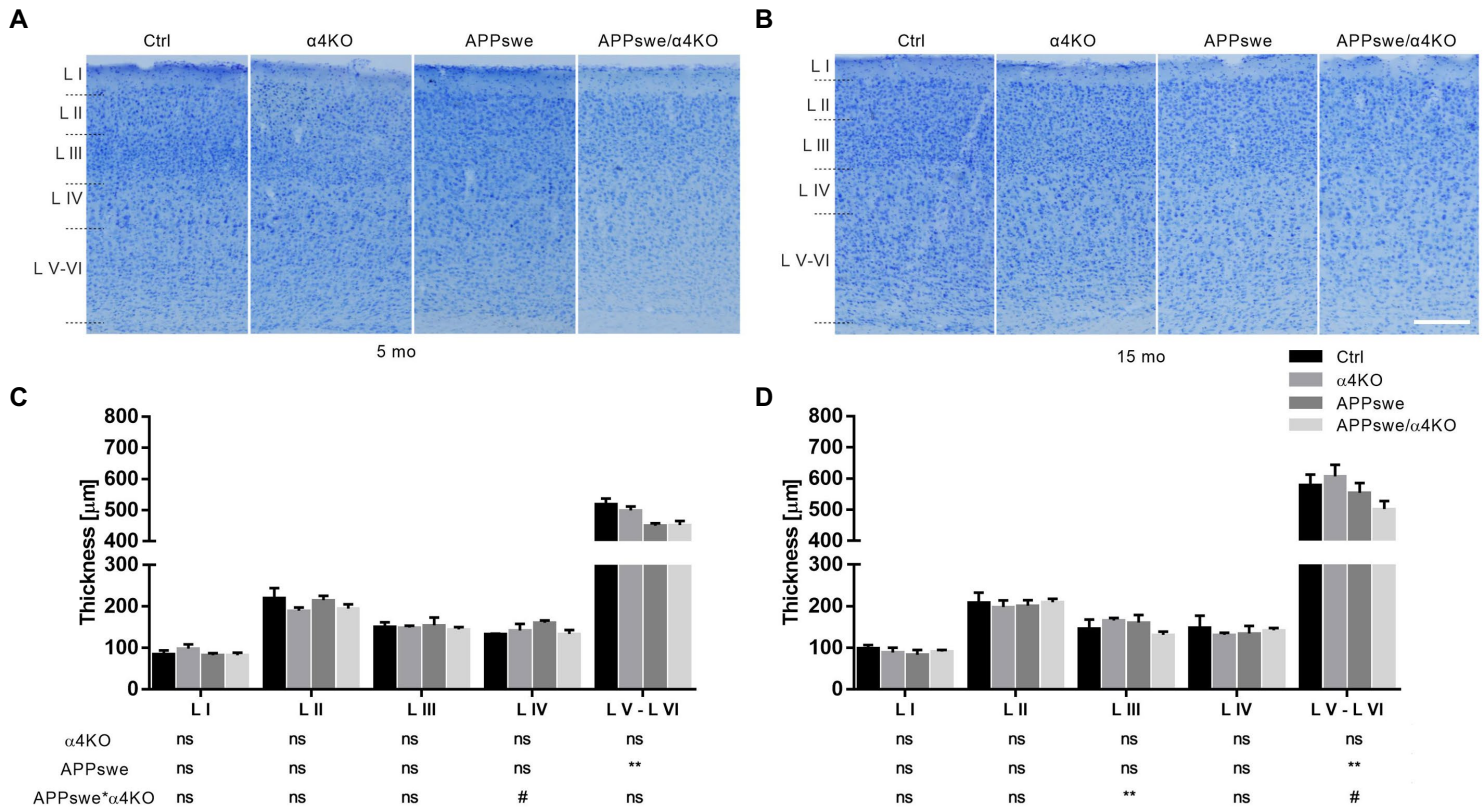
Quantitative analysis of Nissl-stained somatosensory cortex of 5 mo **(A,C)** and 15 mo **(B,D)** mice. Representative images of Nissl staining in layers I–VI of sCtx of 5 mo **(A)** and 15 mo **(B)** Ctrl, α4KO, APPswe, and APPswe/α4KO mice and relative quantification **(C,D)**. Scale bar: 200 μm. Data are shown as mean ± SEM and compared by two-way ANOVA test, ^**^p < 0.01, # 0.10 > *p* > 0.05 as trend for a significant difference. L I–VI, layers I–VI.

In 5 mo mice, a significant reduction was observed in cortical layers V and VI of APPswe mice, while α4KO had no significant effect (Figure 2A).

The same reduction was confirmed in 15 mo mice and restricted to layers V and VI; moreover, a synergistic effect of APPswe and α4KO was shown in layer III (Figure 2B).

As in Tg2576 mice Aβ-related neurodegenerative changes are extended also to the Hip, we analyzed the thickness of pyramidal cell layers in the different Cornu Ammonis (CA) fields.

No significant effect of APPswe or α4KO was observed in 5 mo mice (Supplementary Figure 2A). In 15 mo mice, while APPswe induced a significant decrease in pyramidal cell layer thickness in CA1 field and intermediate part of CA3 field, α4KO had no significant effect on these parameters in any hippocampal subregion analyzed (Supplementary Figure 2B).

Taken together, these results demonstrate that APPswe expression is associated with both cortical and hippocampal atrophy without any substantial effect of α4KO. Detailed statistical reports are shown in Supplementary Table 1.

### 3.3. Synaptophysin immunoreactivity

Synaptic alterations represent an important feature of AD neuropathology and reductions in neuronal processes and synaptic density strongly correlate with cognitive decline in AD. Specifically, it has been reported that elevated SYN ir in cortico-hippocampal regions of aged Tg2576 mice is associated to impaired cognitive functions and possibly to pathophysiologic synaptic processing (King and Arendash, 2002). We tested whether α4* nAChR loss influences APPswe-related increase in SYN ir at cortical and hippocampal levels of aged mice.

**Figure 3:**
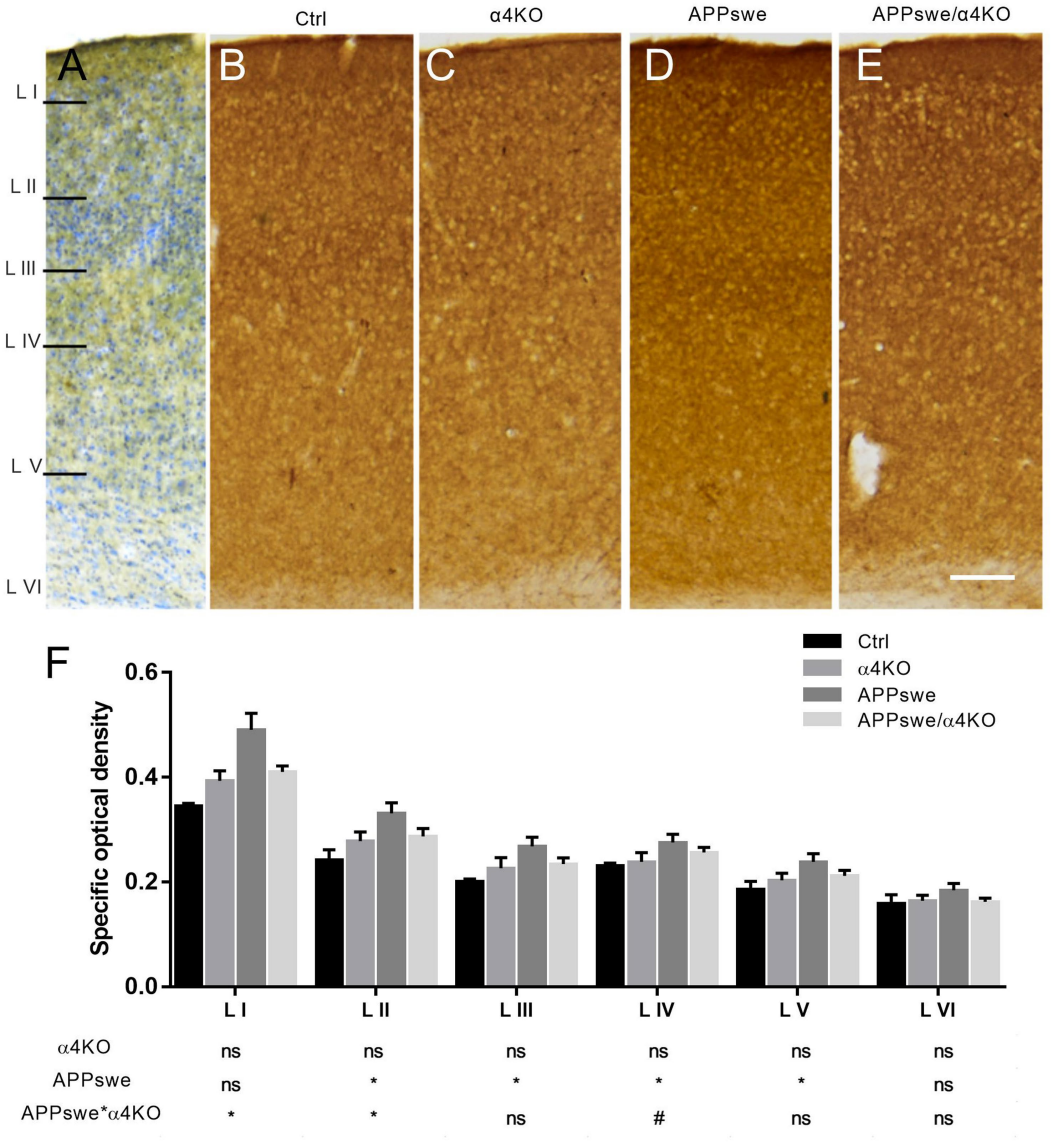
Quantitative analysis of SYN immunoreactivity in the somatosensory cortex of 15 mo mice. **(A–E)** Representative images of SYN ir in layers I-VI of sCtx **(A)** of 15 mo Ctrl **(B)**, α4KO **(C)**, APPswe **(D)**, and APPswe/α4KO **(E)** mice. Scale bar: 200 μm. **(F)** Densitometric analysis of SYN ir in layers I–VI of sCtx. Data are shown as mean ± SEM and compared by two-way ANOVA test, ^*^p < 0.05, # 0.10 > *p* > 0.05 as trend for a significant difference. L I–VI, layers I–VI.

In all experimental groups, SYN ir was particularly intense in both hippocampal and cortical regions. While in 5 mo mice, the densitometric (specific optical density) and morphometric (total area, internal area, length) analyses did not evidence any significant difference between the groups in any brain region analyzed (not shown), in 15 mo mice several regions, such as CA3 field of Hip and cCtx (King and Arendash, 2002), showed alterations in SYN ir in APPswe mice that were partially counteracted by α4* nAChR loss (Figure 3).

At cortical level, the SYN ir appeared widely distributed in layers I-VI, except for the areas occupied by neuronal somata. APPswe induced an increase in SYN ir in the external layers of sCtx (King and Arendash, 2002) that was partially counteracted by α4 loss (Figure 3). No effect either of APPswe or α4* nAChR loss was detected outside cortico-hippocampal regions, such as the caudate-putamen (CPU; not shown).

**Figure 4:**
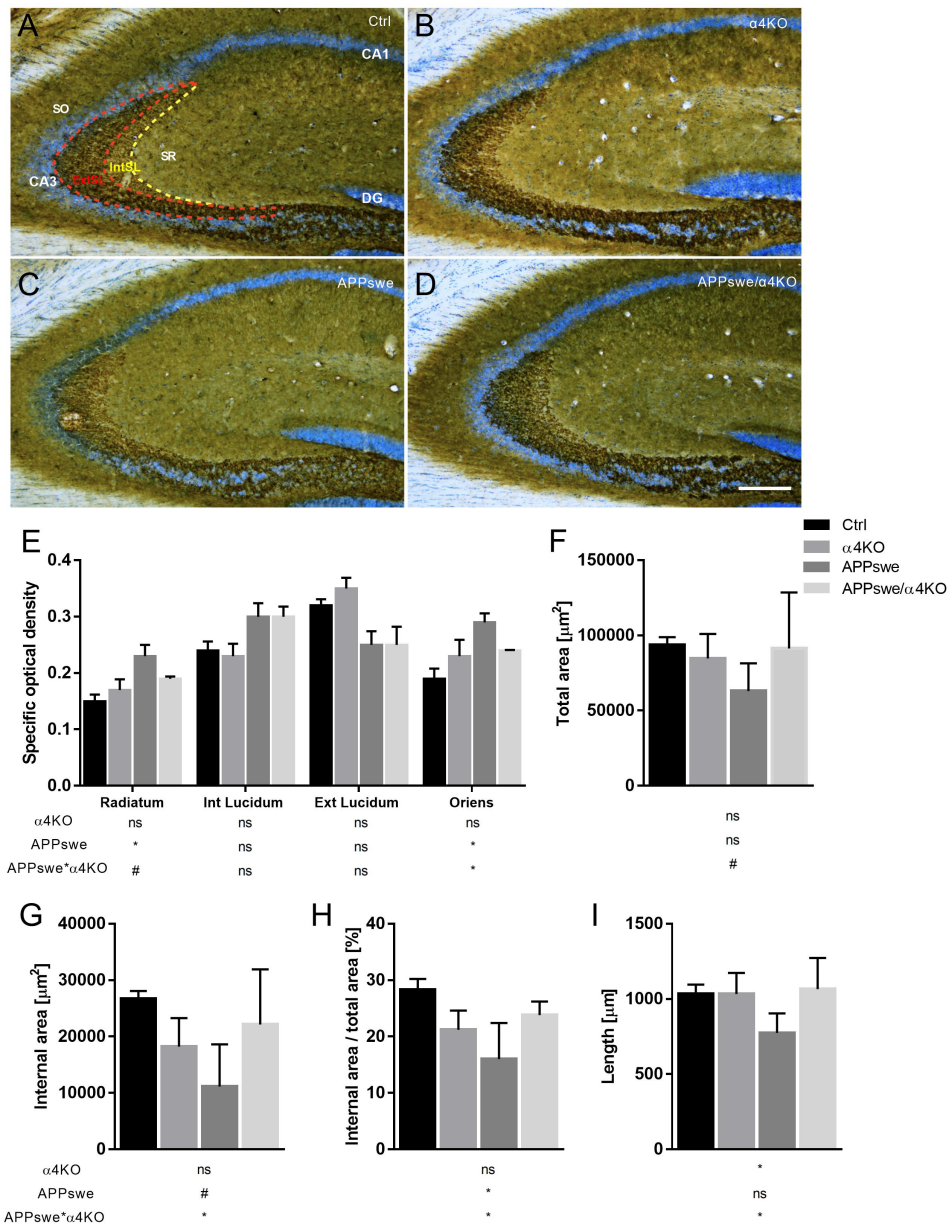
Quantitative analysis of SYN immunoreactivity in hippocampal CA3 field of 15 mo mice. **(A–D)** Representative images of SYN ir in the CA3 field of 15 mo Ctrl **(A)**, α4KO **(B)**, APPswe **(C)**, and APPswe/α4KO **(D)** mice. Scale bar: 200 μm. **(E–I)** Densitometric analysis of SYN ir in hippocampal stratum radiatum, internal (dashed yellow line) and external (dashed red line) stratum lucidum and stratum oriens of CA3 field by analyzing specific optical area **(E)**, total area **(F)**, internal area **(G)**, internal area/total area **(H)**, and length **(I)**. Data are shown as mean ± SEM and compared by two-way ANOVA test, ^*^p < 0.05, # 0.10 > *p* > 0.05 as trend for a significant difference. SR, Stratum Radiatum; SL, Stratum Lucidum; IntSL, internal SL; ExtSL, external SL; SO, Stratum Oriens.

At hippocampal level, the pyramidal cell layer showed very light staining while the areas innervated by mossy fibers showed intense ir. The analysis was focused on the CA3 field (Figures 4A–D). The densitometric analysis showed a significant APPswe-related increase in the intensity of SYN ir in the stratum radiatum (SR) and stratum oriens (SO), but not in the stratum lucidum (SL), that was counteracted by the loss of α4* nAChRs (Figure 4E).

To assess whether changes in the intensity of SYN ir are accompanied by alterations in the shape and/or size of the stained areas, we focused our analysis on the SL of the CA3 field, the subregion with the highest density of SYN+ structures. We found that a number of morphometric parameters, i.e., the area of the internal more intensely stained part of the SL layer, the length of the SL and the ratio between internal and external parts of the SL, were significantly reduced in APPswe mice, alterations that were significantly counteracted by the loss of α4* nAChRs (Figures 4F–I).

Overall, present analysis shows that α4KO counteracts a number of APPswe-associated synaptic alterations, evidenced by significant changes in the intensity and/or distribution of SYN ir, in both cerebral cortex and hippocampus. Detailed statistical analysis is reported in Supplementary Table 1.

### 3.4. Glial fibrillary acidic protein immunoreactivity

**Figure 5:**
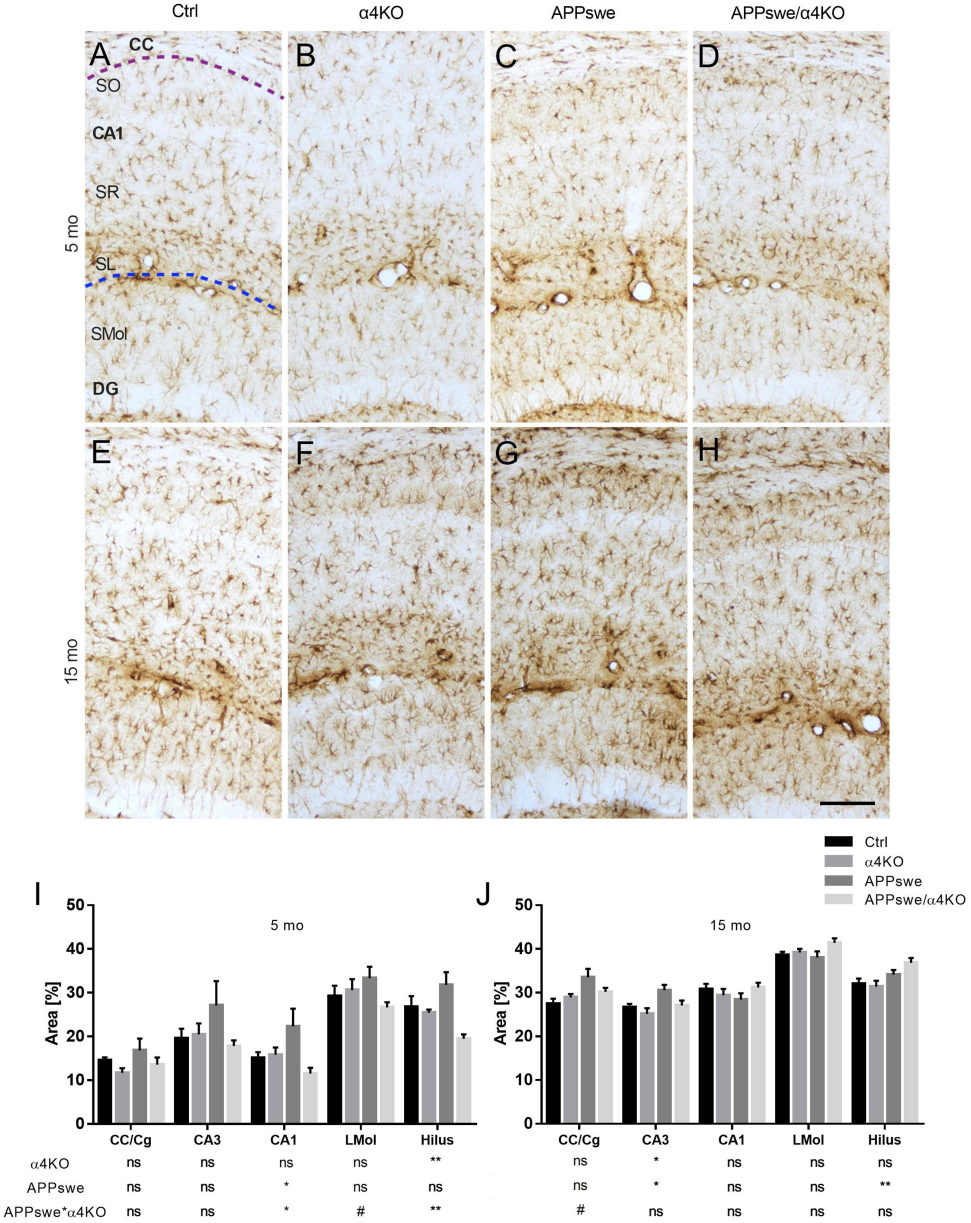
Quantitative analysis of GFAP immunoreactivity in 5 and 15 mo mice. **(A–H)** Representative images of GFAP+ immunoreactivity in 5 mo **(A–D)** and 15 mo **(E–H)** mice. Astrogliosis was analyzed in the Hip of Ctrl **(A,E)**, α4KO **(B,F)**, APPswe **(C,G)**, and APPswe/α4KO **(D,H)** mice. Scale bar = 100 μm. **(I,J)** Quantitative analysis of GFAP ir in CC, Cg and Hip in 5 **(I)** and 15 **(J)** mo mice. Data are shown as mean ± SEM and compared by two-way ANOVA test, ^*^p < 0.05, ^**^p < 0.01, # 0.10 > *p* > 0.05 as trend for a significant difference. CC, corpus callosum; Cg, cingulate cortex; CA1, Cornu Ammonis 1 of hippocampus; LMol, stratum lacunosum-moleculare.

To evaluate how APPswe age-dependently influences astrogliosis, and whether α4KO modulates this process, brain sections from Ctrl, α4KO, APPswe and APPswe/α4KO mice were labeled with anti-GFAP Ab for IHC analysis. Several brain regions were analyzed, i.e., cingulate cortex (Cg), corpus callosum (CC) and hippocampal regions, at both 5 and 15 mo of age (Figure 5).

In 5 mo mice, when there was no evidence for Aβ plaque deposition in any brain region, α4KO significantly counteracted APPswe-related increase in GFAP ir in the hilus of dentate gyrus (DG) and stratum oriens (SO) of the CA1 field while a trend for a significant difference was observed in the stratum lacunosum-moleculare of the CA1 field (Figure 5I).

The same analysis was carried out in 15 mo mice, when Aβ plaque deposition was present in both cortical and hippocampal regions. At this age, APPswe expression was associated with a significant increase in GFAP ir in the hilus of DG, CA3 and the cingulate cortex/medial corpus callosum region (CC/Cg), that was counteracted by α4KO in CA3 field (Figure 5J).

Altogether, these results show that the loss of α4* nAChRs reduces the signs of astrocyte activation that appear already in 5 mo Tg2576 mice, before Aβ plaque deposition, and this effect is maintained at later stages when amyloid neuropathology is extensive.

### 3.5. Ionized calcium-binding adapter molecule 1 immunoreactivity

Similar to astrocytes, morphological changes in microglial cells, such as increased cell soma size and process retraction and thickening, have been observed in both AD and mouse transgenic models of AD (Leyh et al., 2021). The assessment of microglial number and morphological alterations was performed in both 5 and 15 mo mice to reveal whether the loss of α4* nAChRs influences distribution and morphology of microglia in APPswe mice.

**Figure 6:**
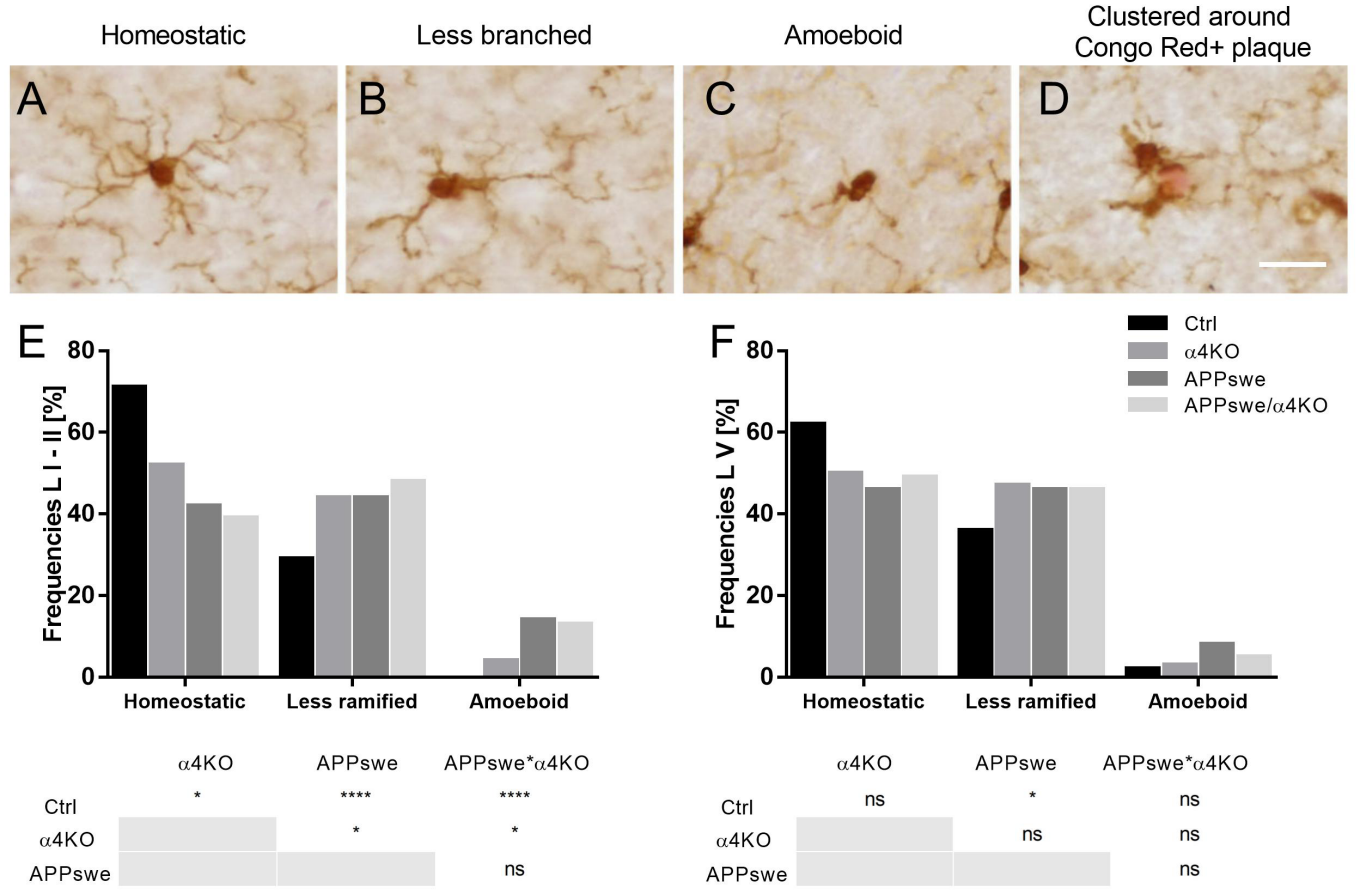
Classification of morphological phenotypes of microglial cells in 5 mo mice. **(A–D)** Representative images of homeostatic **(A)**, less ramified **(B)**, isolated amoeboid **(C)** or clustered around Congo Red+ plaque (in red) Iba1+ microglia. Scale bar = 40 μm. **(E,F)** Quantitative analysis of Iba1+ cell frequency in layer I–II **(E)** and layer V of sCtx of 5 mo mice. Frequencies are shown as % by each group and compared by Chi-square test, ^*^p < 0.05, ^****^p < 0.0001. L I–II, V, layers I–II, V.

The stereological analysis showed that both APPswe and α4KO induced a number of changes already in 5 mo mice in sCtx. In fact, while the number of Iba1+ cells was not different between the groups (Supplementary Figures 3A–F), the prevalence of the three principal microglial subclasses, i.e., homeostatic, less ramified and amoeboid cells, was significantly altered by both APPswe and α4KO in layer I-II and by APPswe in layer V (Supplementary Figure 4; Figures 6A–F).

Further analysis of the microglial subpopulations in layers I–II showed that APPswe induced an increase in the number of amoeboid and a decrease in the number of homeostatic cells as well as an increase in the perimeter and a decrease in the number of processes of activated cells, whereas α4KO induced a decrease in the number of processes of Iba+ cells (Supplementary Figure 4). In layer V, APPswe increased the area of less ramified cells and the perimeter of homeostatic cells, whereas α4KO increased both area and perimeter of less ramified cells (Supplementary Figure 5). Overall, both APPswe and, to a lower extent, α4KO were associated to a reduced number of homeostatic microglial population.

**Figure 7:**
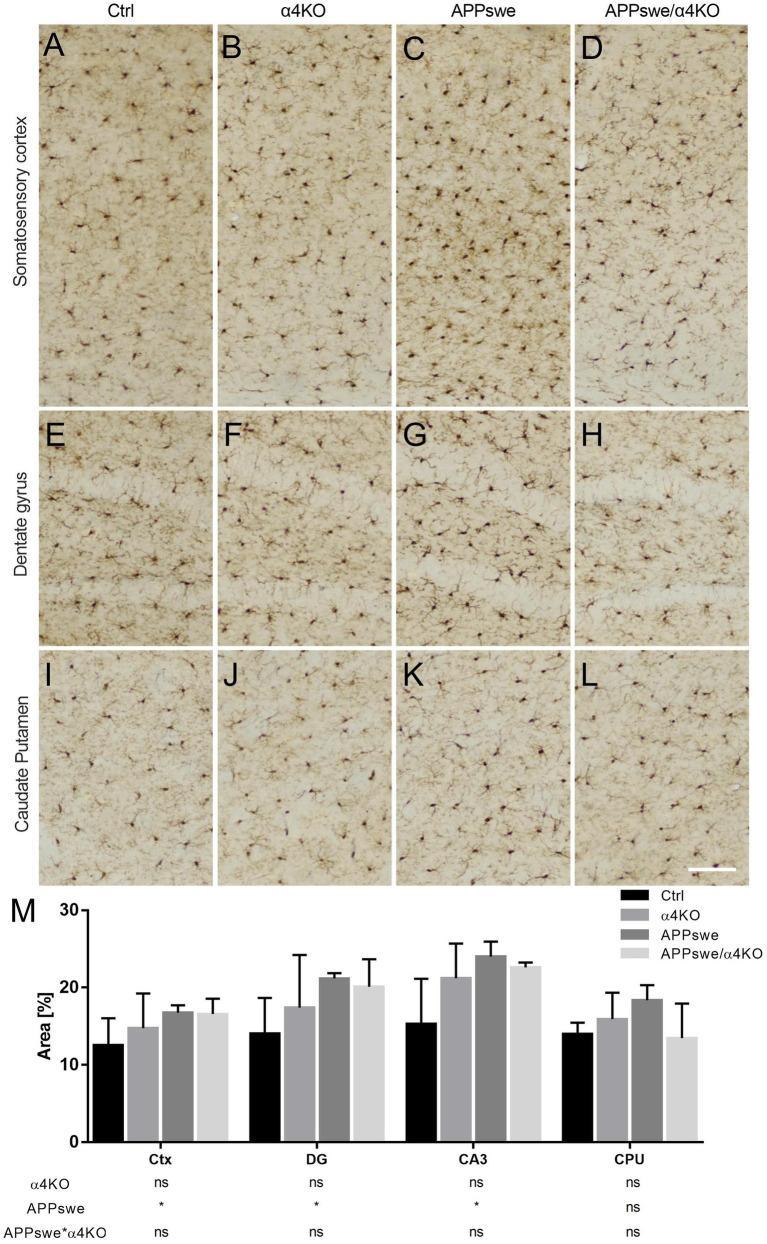
Quantitative analysis of Iba1 immunoreactivity in 15 mo mice. **(A–L)** Representative images of Iba1+ staining through the sCtx **(A–D)**, hippocampal DG **(E–H)** and CPU **(I–L)** of Ctrl **(A,E,I)**, α4KO **(B,F,J)**, APPswe **(C,G,K)**, and APPswe/α4KO **(D,H,L)** 15 mo mice. Scale bar = 100 μm. **(M)** Quantitative analysis related to % area coved by Iba1+ ir in sCtx, DG and CA3 field of hippocampus, and CPU Data are shown as mean *±* SEM and compared by two-way ANOVA test, ^*^p < 0.05. Ctx, cerebral cortex; DG, dentate gyrus; CA3, Cornu Ammonis 3 of hippocampus; CPU, caudate putamen.

Several changes were also observed in 15 mo mice. We first performed an analysis of the number of Iba1+ cells in several brain regions. APPswe expression significantly increased the number of Iba1+ cells in sCtx (Figures 7A–D), DG (Figures 7E–H), and the CA3 field (not shown) but not in the CA1 field (not shown) and CPU (Figures 7I–L). α4KO did not induce any significant change in any brain region examined (Figure 7M).

**Figure 8:**
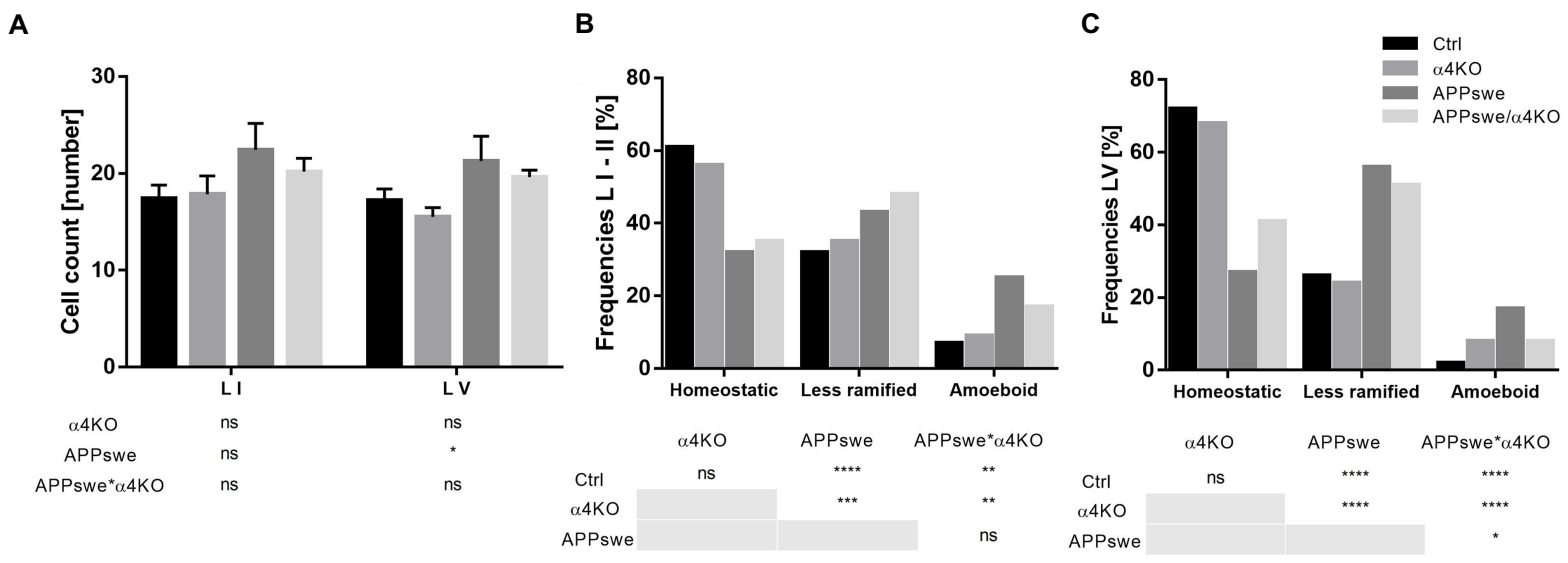
Classification of morphological phenotypes of microglial cells in 15 mo mice. Quantitative analysis of microglial density in layers I–II and layer V **(A)** of sCtx and relative frequency in homeostatic, less ramified, or amoeboid state **(B,C)**. In **A**, data are shown as mean *±* SEM and compared by two-way ANOVA test; in **B,C**, frequencies are shown as % by each group and analyzed by Chi-square test. ^*^p < 0.05, ^**^p < 0.01, ^***^p < 0.001, ^****^p < 0.0001. L I–II, V, layers I–II, V.

Then we performed a stereological analysis of Iba1+ cells in the layers I–II and V of the sCtx by using quantitative parameters like soma area and perimeter, process length and number of processes. In layers I–II, APPswe expression did not significantly alter total cell number (Figure 8A), significantly decreased the number of processes/cell without changing process length, and increased the size of Iba1+ microglial cells, while α4KO did not induce any significant change in Iba1+ cells (Supplementary Figure 6).

In layer V, APPswe expression significantly increased the number and the size of Iba1+ microglial cells (Figure 8A), and decreased their number of processes/cell without changing process length, while α4KO did not induce any significant change in Iba1+ cells (Supplementary Figure 7).

The morphological analysis of homeostatic, less ramified and amoeboid cells demonstrated a shift toward less ramified and amoeboid Iba1+ cells in APPswe mice with respect to Ctrl or α4KO mice, that was partially reverted in APPswe/α4KO mice in layer V but not in layers I–II (Figures 8B,C).

The analysis of the morphometric features of the three classes of microglia showed similar changes in layers I–II and layer V, though more intense in layer V. APPswe expression was associated with a significant increase in the number and size of amoeboid and reactive microglia, this latter showing also a decreased number of processes, and a significant decrease in cell and process number and increase in size of resting microglia in layer I–II and, more intensely, in layer V. These alterations in microglia populations were not significantly modified by α4KO (Supplementary Figure 4).

In conclusion, while α4KO was not able to significantly counteract the morphological changes induced by APPswe expression in microglia, it could partially counteract the shift toward more reactive forms in layer V, in parallel with a reduction in plaque load (see above). Detailed statistical analysis is reported in Supplementary Table 1.

## 4. Discussion

The present study reports a number of protective effects of the loss of α4* nAChRs on the neuropathological alterations that develop over time in Tg2576 (APPswe) mice, a widely studied mouse model of AD that expresses a human APP transgene carrying the amyloidogenic Swedish mutation.

Our data clearly demonstrated that at 15 months of age, APPswe mice show diffuse Aβ deposition in corticohippocampal regions, such as nCtx, Ent, Hip (Figure 1), an atrophy of neocortical internal layers (Figure 2) and hippocampal CA1 and CA3 pyramidal layers (Supplementary Figure 2), an increase in the intensity of SYN ir in the external nCtx (Figure 3) and some hippocampal layers (Figure 4), GFAP+ astrogliosis in cortico-hippocampal white and gray matter (Figure 5) and Iba1+ microgliosis in corticohippocampal regions (Figure 7).

In the context of these complex and diffuse neuropathological changes in corticohippocampal regions induced by constitutive expression of APPswe, the loss of α4* nAChRs exerted some protective effects. The principal effect of α4KO was to markedly reduce the load of Aβ plaques in neocortical areas. The plaques were reduced in number but their size distribution was unchanged, suggesting that fewer plaques are initially formed, but once they are seeded their maturation is not altered by the loss of α4* nAChRs. The evidence that hAPP ir in 5 mo mice was not significantly altered by α4KO (Supplementary Figure 1) indicates that loss of α4* nAChRs does not alter APP production, and supports the hypothesis that α4ΚΟ affects APP processing.

In addition, α4KO partially rescued APPswe-induced alterations in corticohippocampal nerve terminals and neuroinflammation, as witnessed by the reduced proportion of the less ramified forms of microglial cells in cortical regions. Moreover, α4KO partially rescued APPswe-induced astrogliosis in corticohippocampal regions. α4KO effects on astrogliosis were detected already at 5 months of age, suggesting that α4* nAChRs may have a detrimental effect on the development of Aβ pathology at early stages when no Aβ plaques or morphological neuronal alterations are detectable.

The impact of α4β2 nAChR deletion on Aβ pathology *in vivo* has been insufficiently investigated up to now. Yet, present evidence for α4KO-associated neuroprotection tallies well with previous studies showing that genetic deletion of β2* nAChR caused reduced intracellular Aβ accumulation in dentate gyrus and improved cognitive functions in hAPP-SLA mice (Lombardo et al., 2016) and improved spatial reference memory in APP/PS1 Tg mice (George et al., 2021). Overall, the accumulating evidence on α4 or β2 KO protection in Tg AD models is at odds with the multiple studies on the detrimental effects of β2* nAChR loss on several types of lesion and neurodegeneration (Zoli et al., 1999; Laudenbach et al., 2002; Bao et al., 2005; Zanardi et al., 2007; Huang et al., 2011; Konsolaki and Skaliora, 2015). Accordingly, in the present study, we observed some evidence for increased microgliosis at 5 months of age in α4KO mice (Figure 6), though not at 15 months of age when α4* nAChR loss became detrimental (Figure 8). Therefore, present and previous evidence points to a specific detrimental role of α4β2 nAChRs in the development of Aβ pathology (discussed below) that may overwhelm their multiple, though still poorly characterized, protective actions observed in other models.

While recent research has been especially focused on the binding and functional interactions between Aβ and α7 nAChRs (George et al., 2021), it has also been clearly shown that different forms of Aβ can bind heteromeric nAChRs including the α4β2 subtype (Wu et al., 2004; Mura et al., 2012). In different experimental conditions and isoforms, Aβ has different effects on α4β2 nAChRs (Jurgensen and Ferreira, 2010). A current interpretation is that increased Aβ concentration in pathological conditions switches the physiological nAChR activation to inhibition and toxicity (Lombardo and Maskos, 2015). Interestingly, it has been shown that expression of α4β2 nAChRs sensitizes the neurotoxicity elicited by oligomeric Aβ (Arora et al., 2015, 2020).

A second line of evidence linking α4β2 nAChRs to Aβ concerns the characterization of Acetyl-His-Ala-Glu-Glu-Amide (HAEE), a peptide corresponding to the sequence of the α4 subunit complementary to the ^11^EVHH^14^ amino acids of Aβ. HAEE analogs block inhibition of α4β2 nAChRs expressed in *Xenopus laevis* oocytes (Mediannikov and Morozov, 2013; Barykin et al., 2020) and zinc-induced dimerization of the Aβ metal-binding domain, thus slowing Aβ aggregation *in vitro* (Tsvetkov et al., 2015; Kozin et al., 2018b). Interestingly, *in vivo* administration of HAEE analogs decreases the number of Congo Red+ plaques in the APPswe/PSEN1dE9 (Tg) AD mouse model. These studies have led to the hypothesis that α4β2 nAChRs through the HAEE-mediated binding of Aβ may serve as seed for the development of amyloid aggregates (Kozin et al., 2018a).

Present evidence supports the hypothesis that α4* nAChRs are directly involved in the seeding of the plaques without influencing plaque maturation and specific Aβ isoform processing. In fact, on the one hand, α4ΚΟ decreases Aβ plaque number, but, on the other hand, it does not change the size distribution of the plaques, thus indicating that α4* nAChRs favor plaque formation but once the plaques are formed do not affect their subsequent fate in a critical way. Reduced amyloid plaque deposition may also result from an accelerated Aβ phagocytosis by microglia, though this hypothesis is not supported by present evidence of maintained or reduced microglial activation in APPswe/α4KO mice.

The evidence that α4* nAChR loss may protect against Aβ pathology adds an important new element to the pathophysiological role of nAChRs in AD. α4β2 nAChRs are markedly decreased in AD brain and this loss is thought to contribute to AD-related cognitive deficits. Accordingly, cholinergic and, as a consequence, nAChR potentiation is the target of current anti-AD pharmacological therapies. It would be of interest to investigate whether and in which way cholinergic drugs interfere with the reciprocal Aβ-α4β2 nAChR interactions that present and previous data highlight.

In conclusion, results of the present investigation indicate that the absence of α4* nAChRs in APPswe mice has a protective effect against Aβ pathology. Further studies are needed to better understand the molecular mechanisms by which α4* nAChRs mediate Aβ-associated neurotoxicity and whether/how these heteromeric receptors are involved in microglial phagocytosis of Aβ.

## Data availability statement

The original contributions presented in the study are included in the article/Supplementary material, further inquiries can be directed to the corresponding author.

## Ethics statement

The animal study was reviewed and approved by Animal Health and Care of the University of Modena and Reggio Emilia (protocol number: 102/2011-B).

## Author contributions

BR and MB: mouse breeding, *in vivo* investigation, and data collection. AV and MZ: data collection and curation, formal analysis, and study supervision. SP and UM: α4KO mouse generation and contribution to study design. MZ: original draft preparation. AV and UM: writing—review and editing. All authors contributed to the article and approved the submitted version.

## Funding

This work was supported by the MIUR Dipartimenti di Eccellenza 2018–2022 and the Unimore-FAR (Competitive projects) to MZ, AV, and MB and the Fondation Vaincre Alzheimer, Fondation Alzheimer, Equipe FRM to UM.

## Conflict of interest

The authors declare that the research was conducted in the absence of any commercial or financial relationships that could be construed as a potential conflict of interest.

## Publisher’s note

All claims expressed in this article are solely those of the authors and do not necessarily represent those of their affiliated organizations, or those of the publisher, the editors and the reviewers. Any product that may be evaluated in this article, or claim that may be made by its manufacturer, is not guaranteed or endorsed by the publisher.

## Supplementary material

The Supplementary material for this article can be found online at: https://www.frontiersin.org/articles/10.3389/fnins.2023.1097857/full#supplementary-material
