# Supplementary figures and images for: Evidence for a protective effect of the loss of α4-containing nicotinic acetylcholine receptors on Aβ-related neuropathology in Tg2576 mice
Source: Front Neurosci. 2023 Apr 11;17:1097857. doi: 10.3389/fnins.2023.1097857 (PMC10126303; doi:10.3389/fnins.2023.1097857)

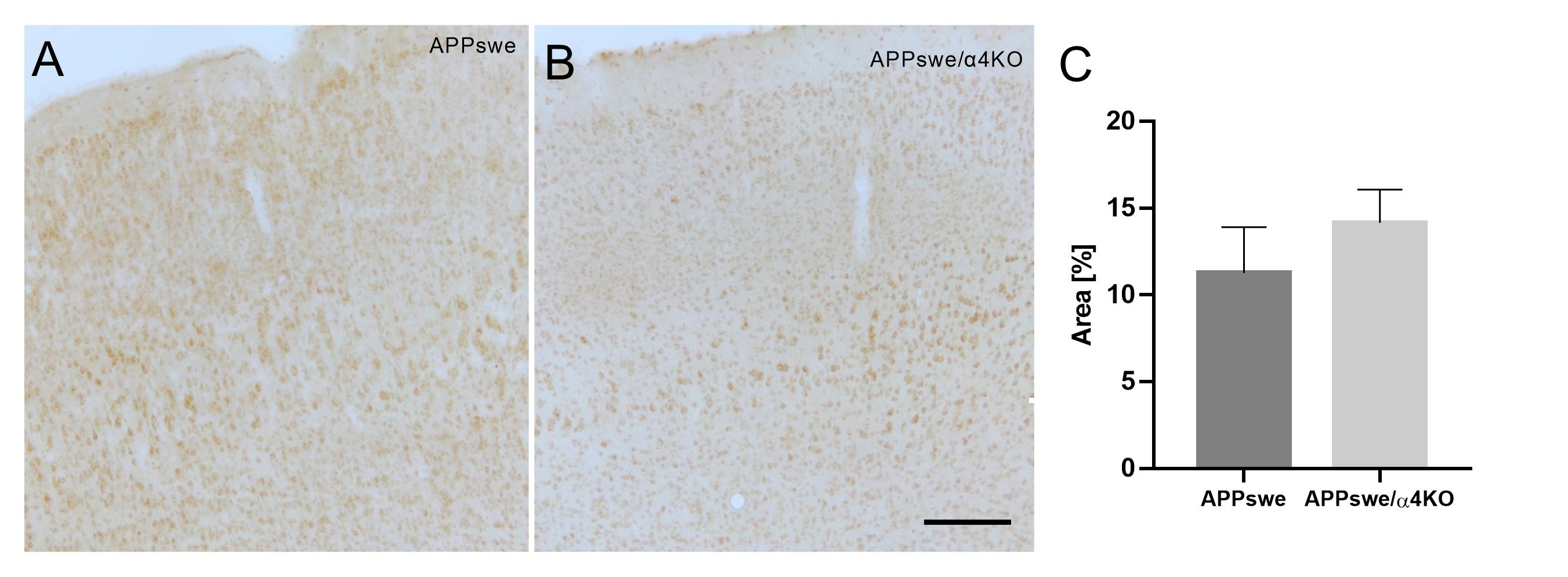

Supplement: Supplementary file 1 [file Image_1.jpg]

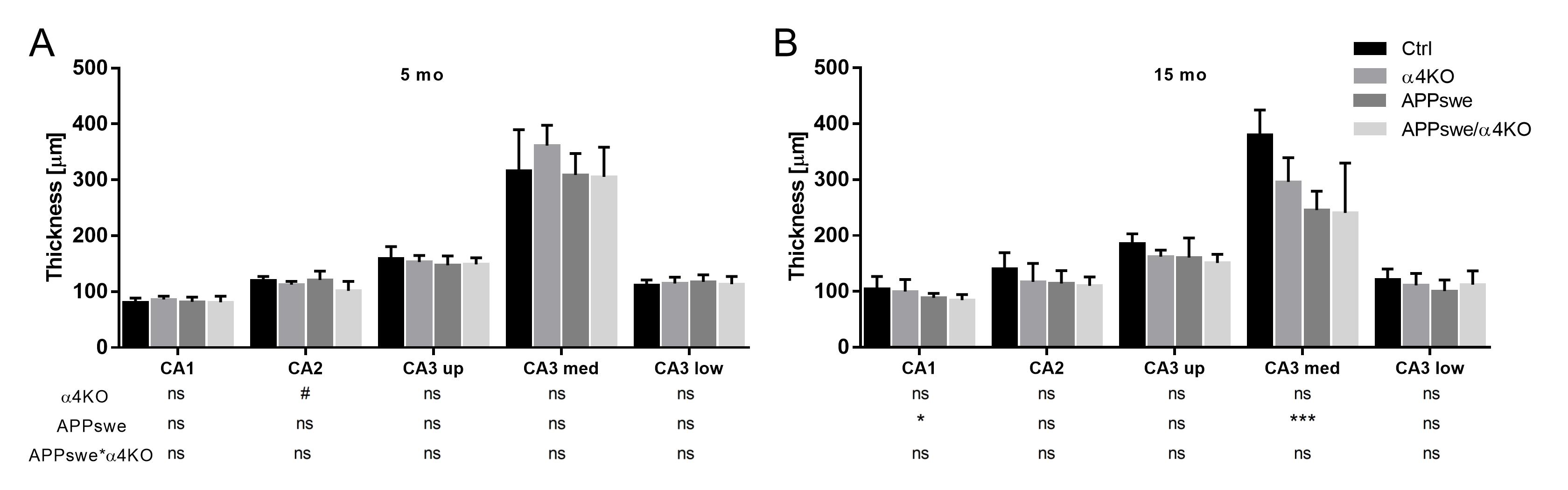

Supplement: Supplementary file 2 [file Image_2.jpg]

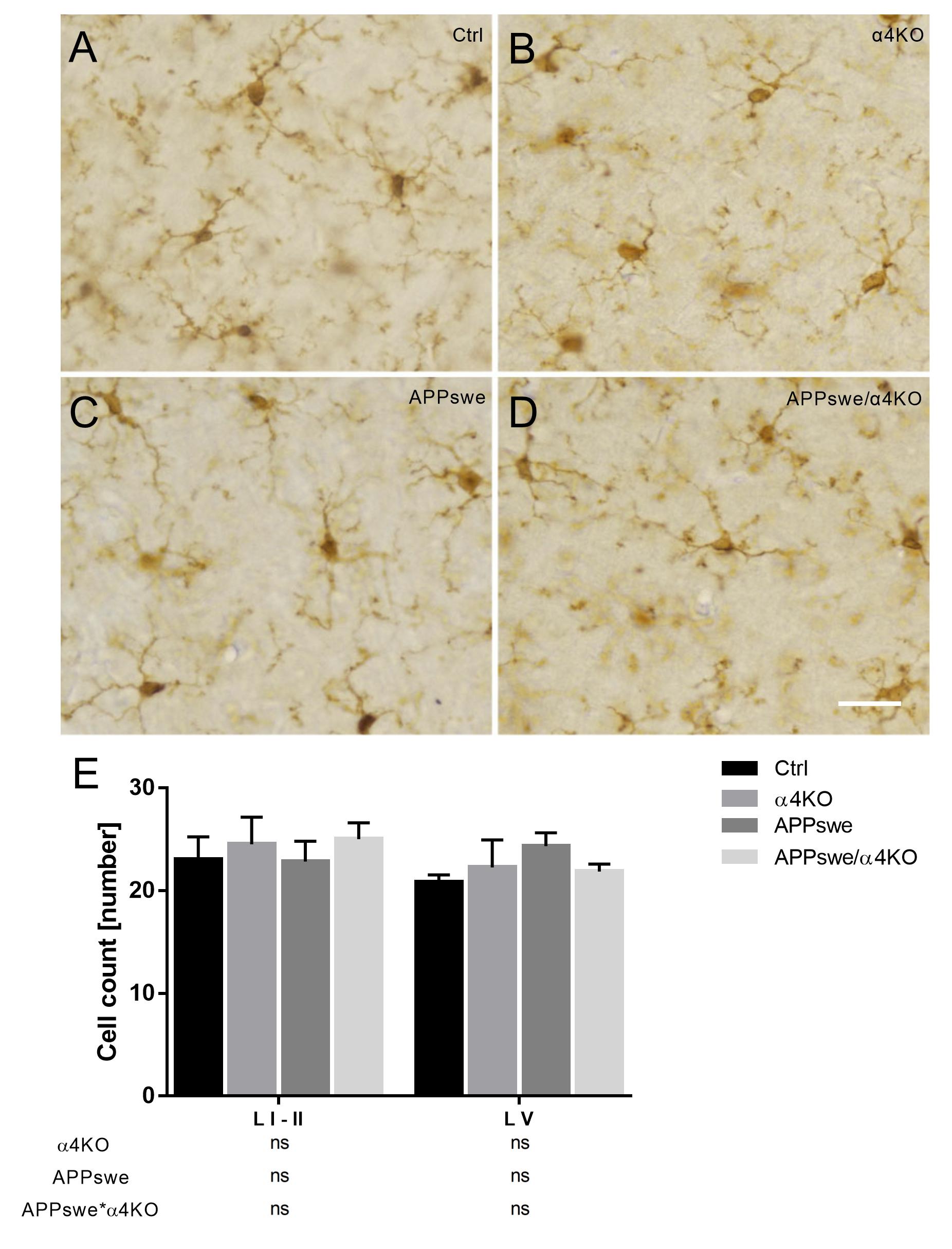

Supplement: Supplementary file 3 [file Image_3.jpg]

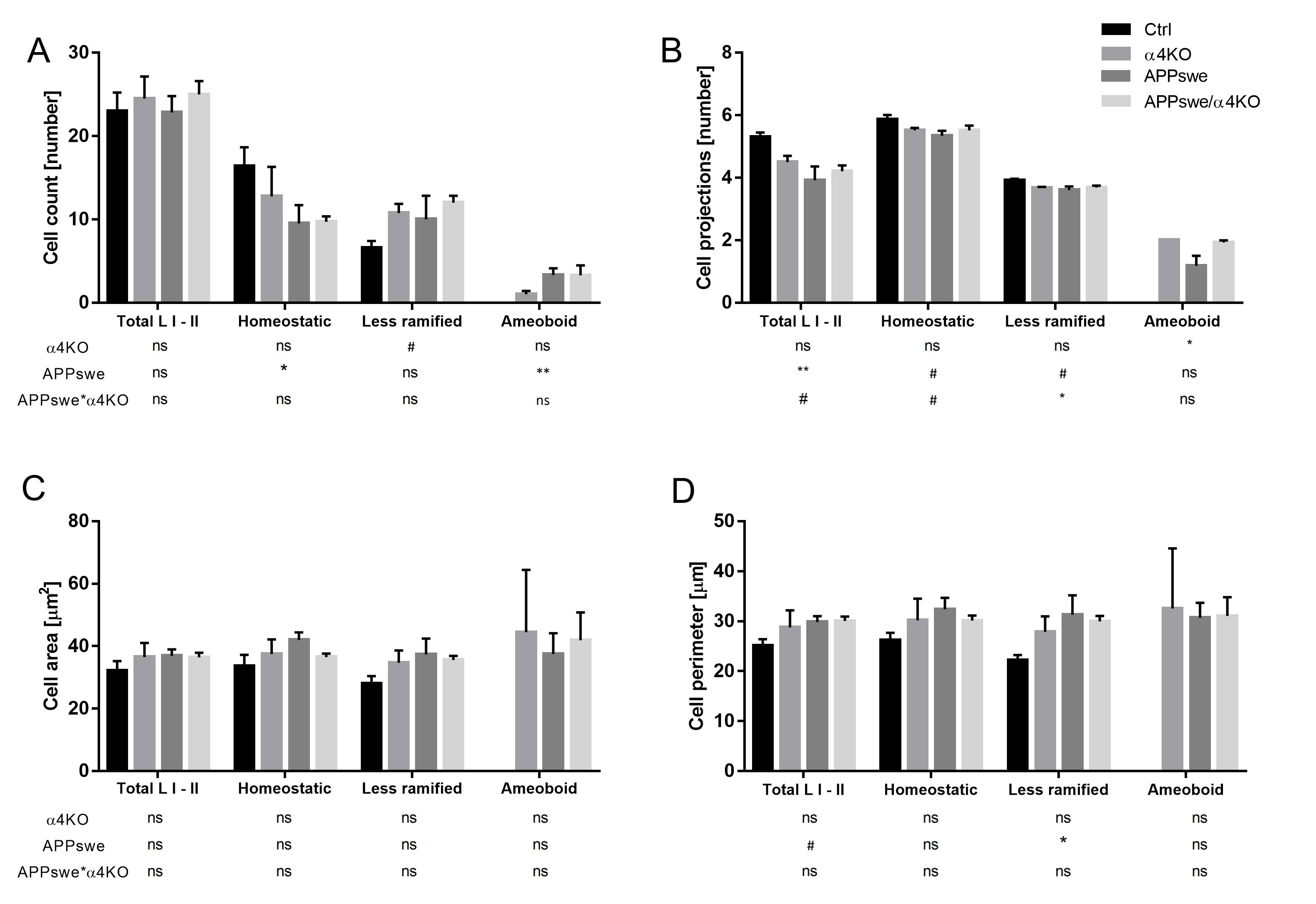

Supplement: Supplementary file 4 [file Image_4.jpg]

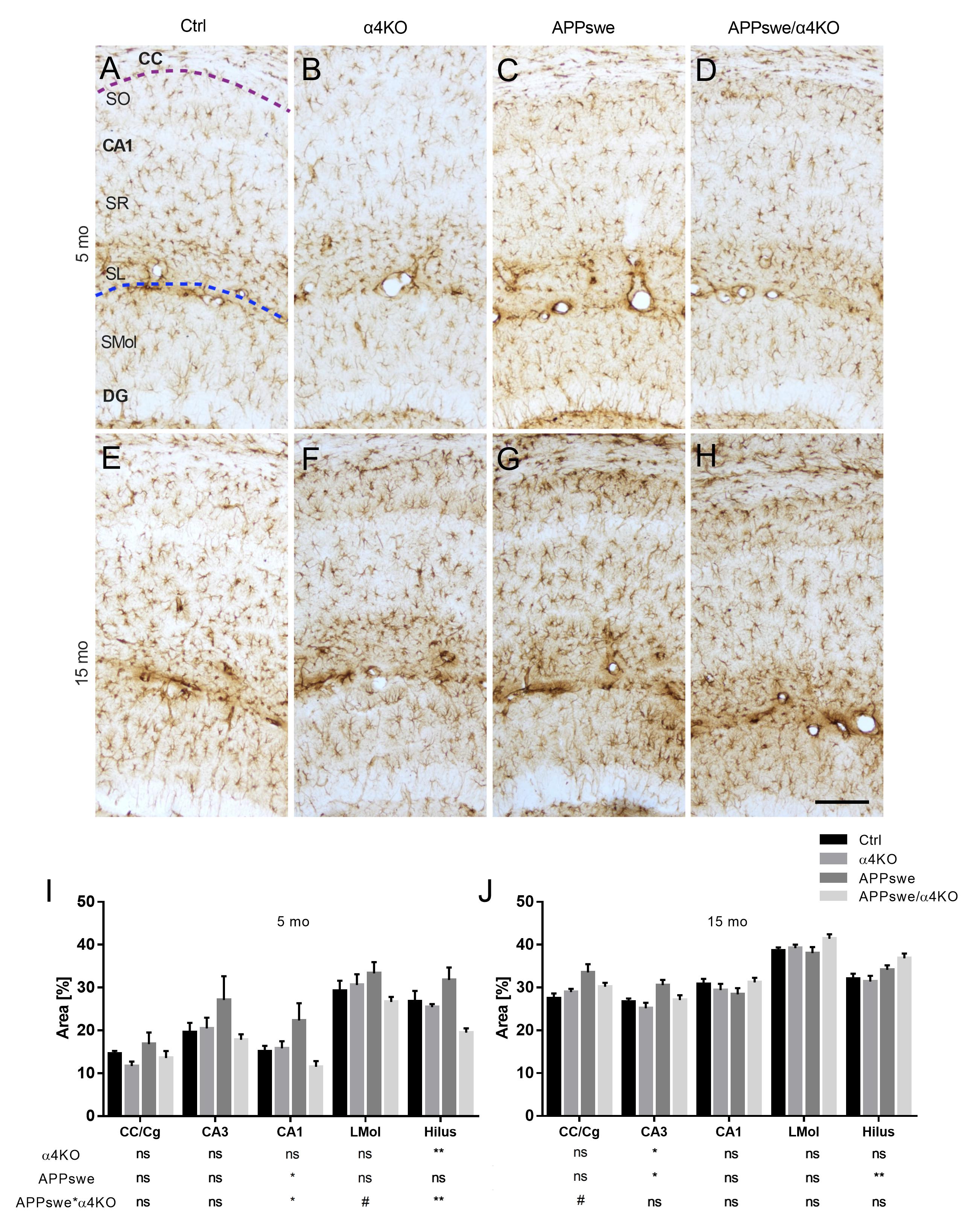

Supplement: Supplementary file 5 [file Image_5.jpg]

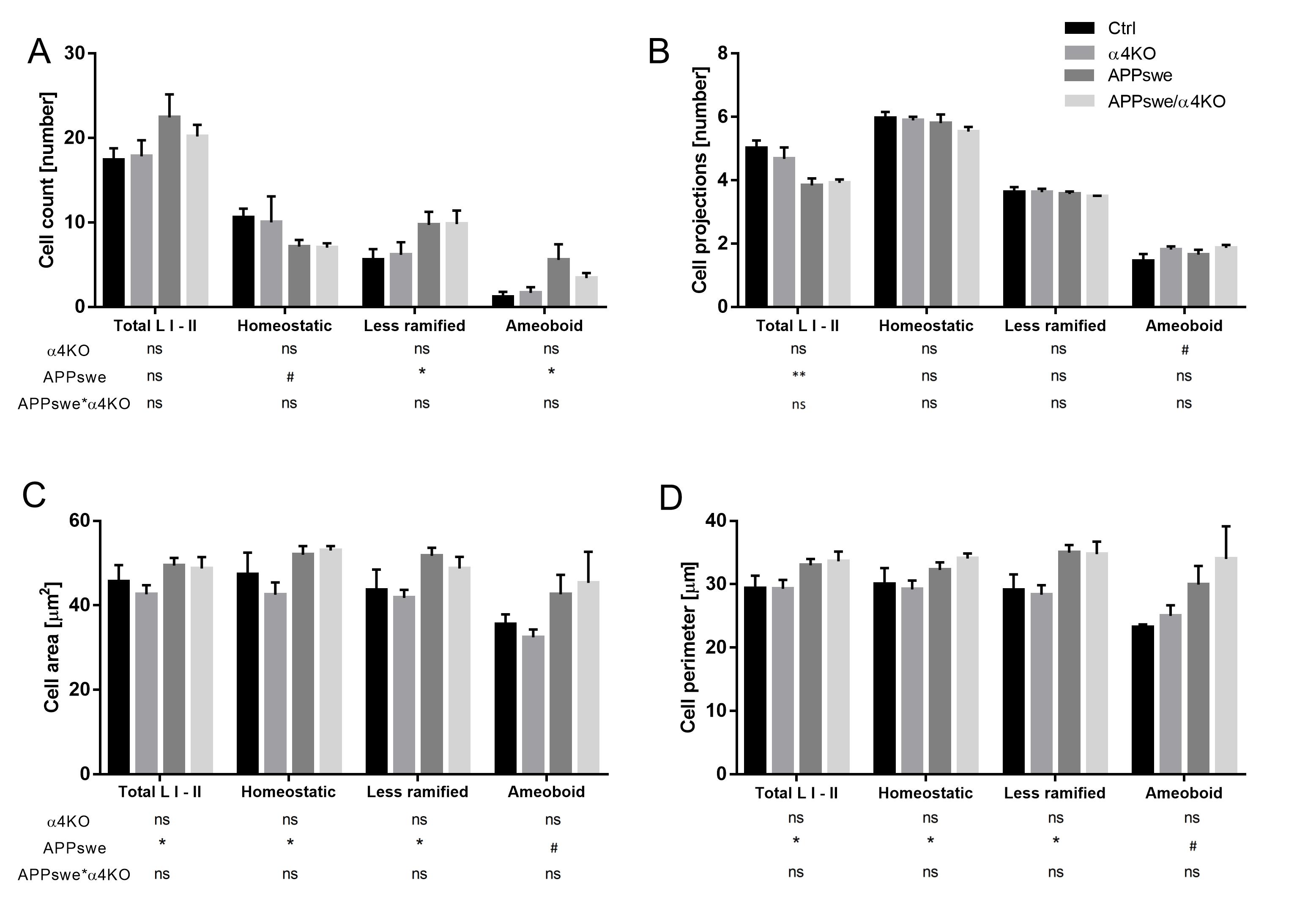

Supplement: Supplementary file 6 [file Image_6.jpg]

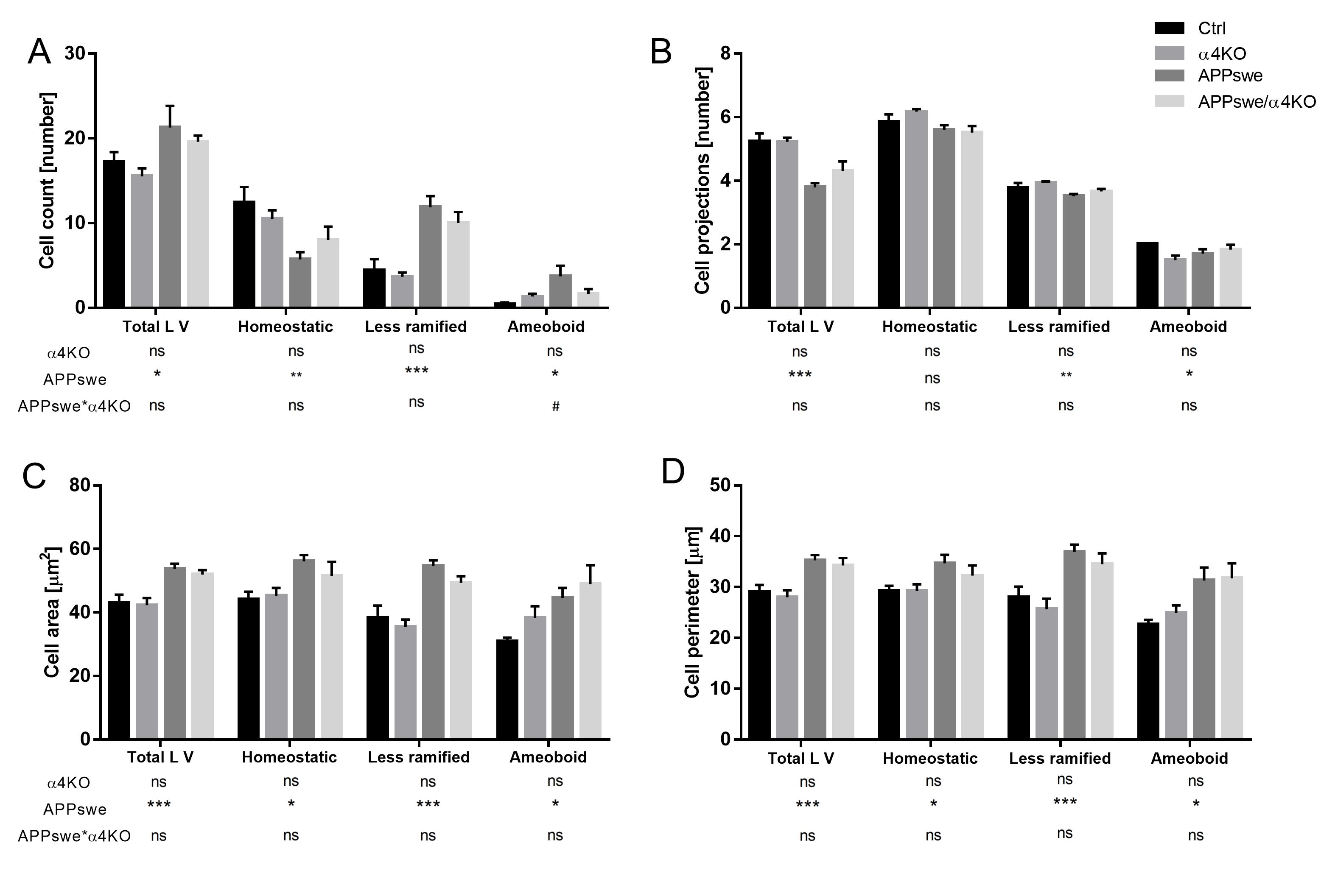

Supplement: Supplementary file 7 [file Image_7.jpg]
